# Supplementary material for: Macrocycle as a “Container” for Dinitramide Salts
Source: Materials (Basel). 2022 Oct 7;15(19):6958. doi: 10.3390/ma15196958 (PMC9570699; doi:10.3390/ma15196958)
Supplement: Supplementary file 1 [file materials-15-06958-s001.zip › materials-1886444-supplementary.pdf]

*Supplementary Materials*

# Macrocycle as a “Container” for Dinitramide Salts

Sergey G. Il'yasov,* Vera S. Glukhacheva, Dmitri S. Il'yasov, and Egor E. Zhukov

*Laboratory for High-Energy Compounds Synthesis, Institute for Problems of Chemical and Energetic Technologies, Siberian Branch of the Russian Academy of Sciences (IPCET SB RAS), Biysk 659322, Altai Krai, Russia*

*[*]Corresponding author. E-mail: ilysow@ipcet.ru (S. Il'yasov)*

**Table of Contents**

| | |
|---|---|
| IR spectra of **7** and **8** | S2 |
| DSC and TGA of **7** and **8** | S3–S5 |
| Testing of compound **8** for burning rate of $KNO_3$/Zr pyrotechnic composition | S6–S7 |
| Testing of compound **8** for burning rate of $KClO_4$/Al pyrotechnic composition | S8–S9 |

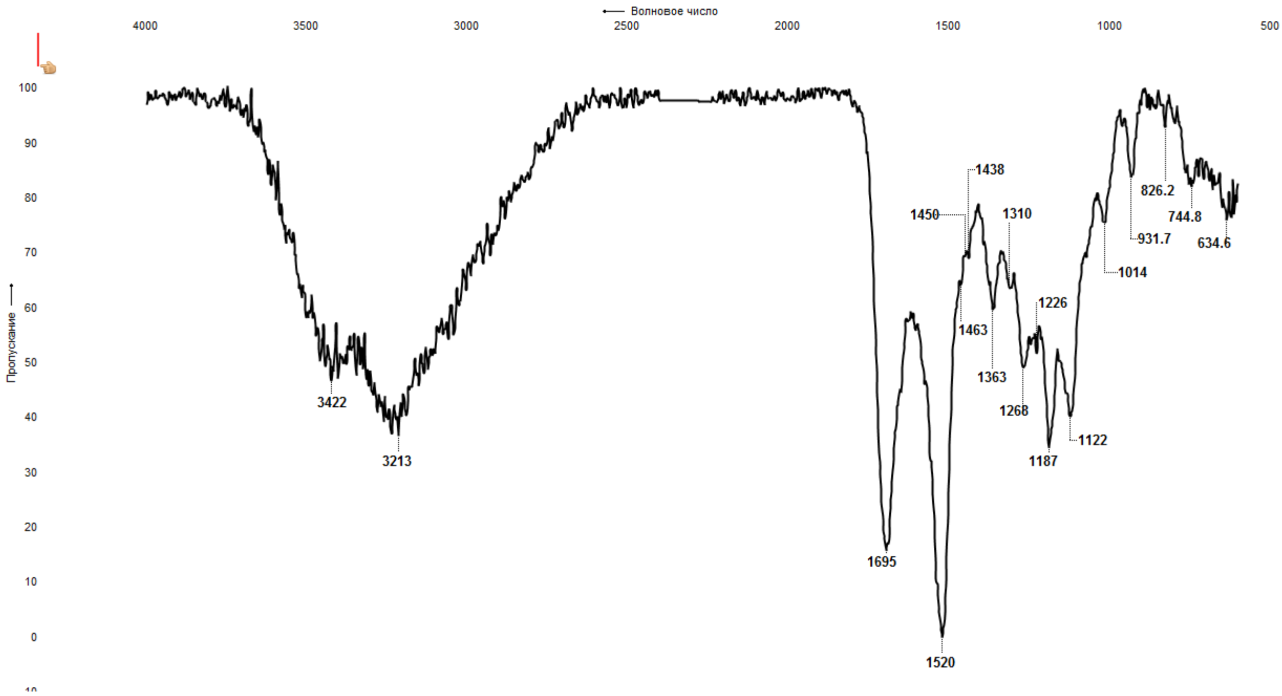


**Figure S1.** IR spectrum of **7**

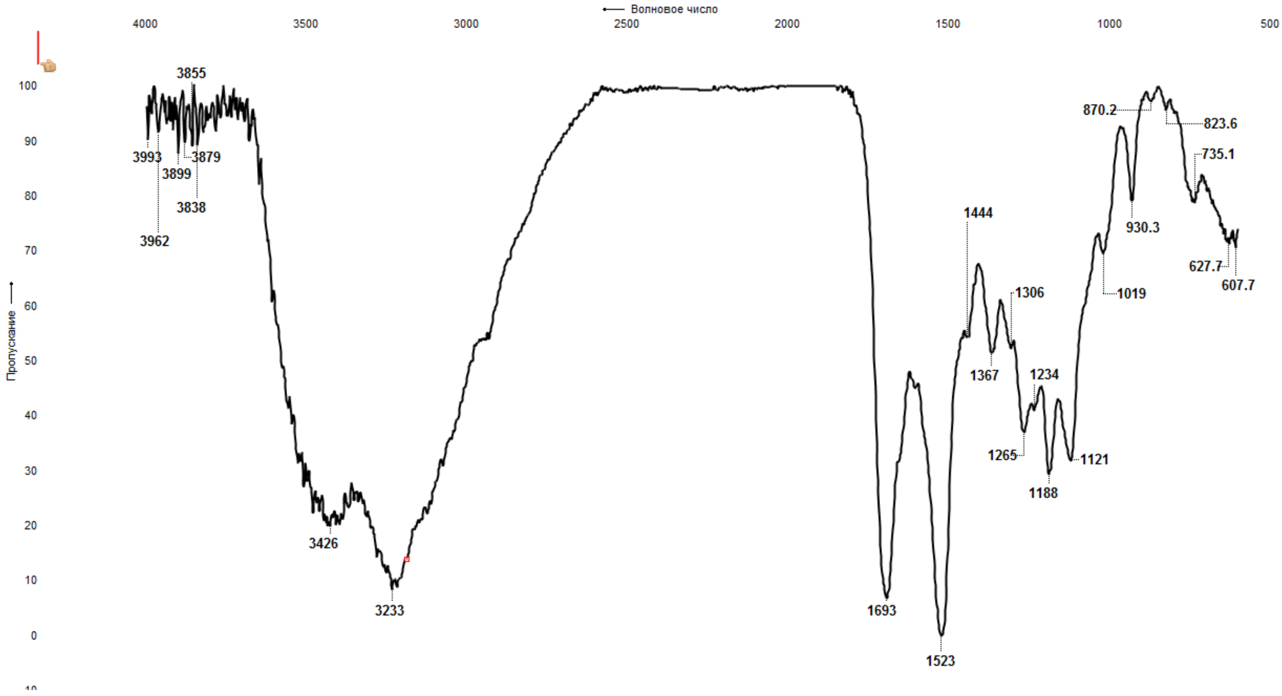


**Figure S2.** IR spectrum of **8**

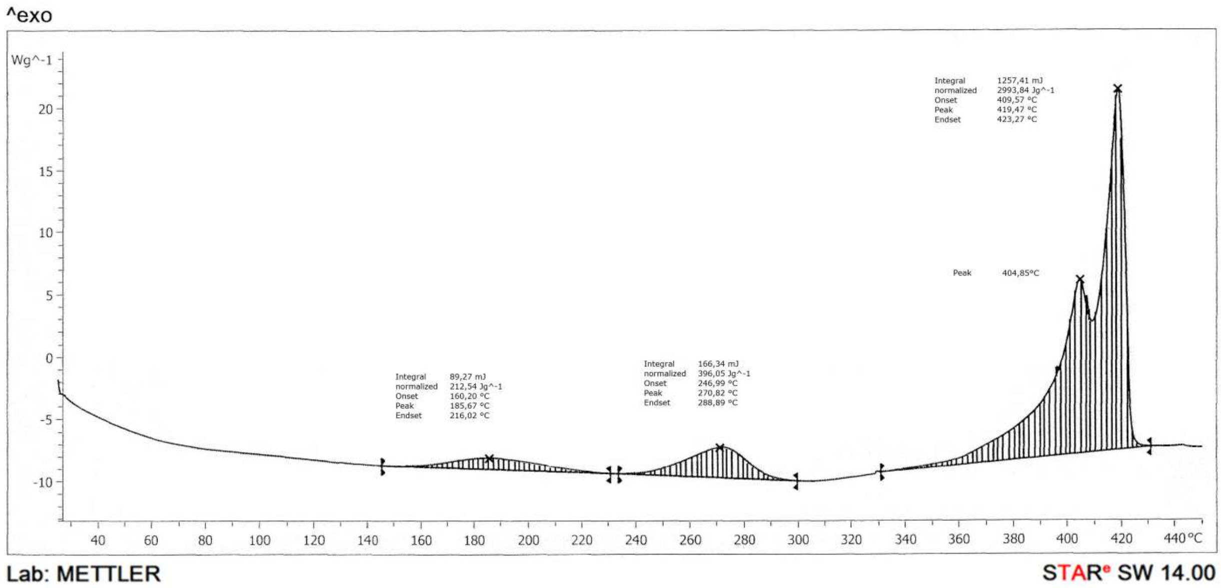


**Figure S3.** DSC of **7**

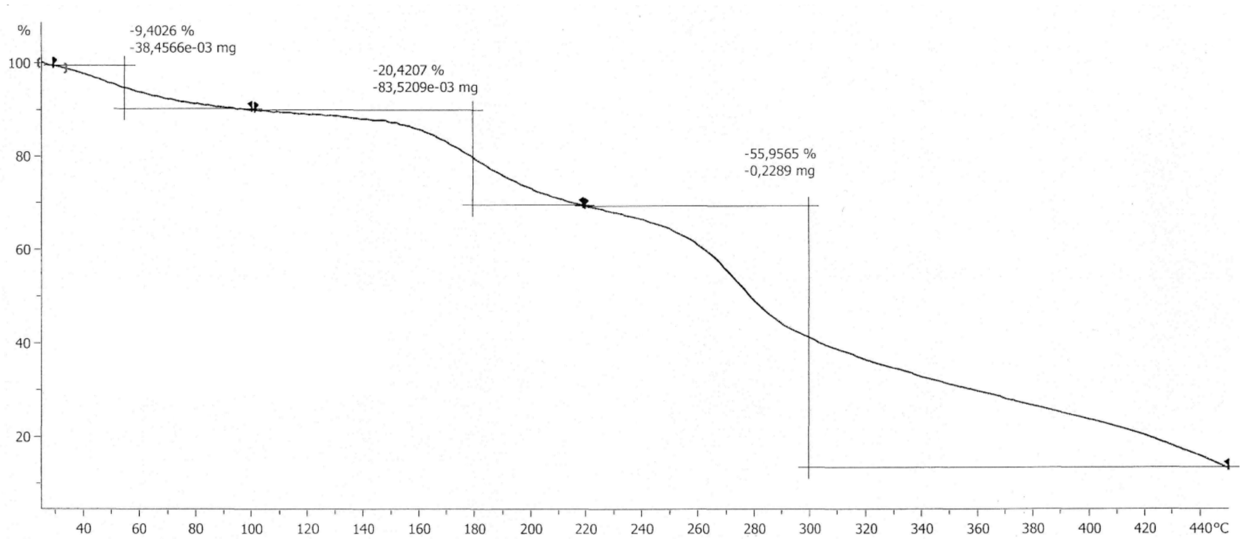


**Figure S4.** TGA of **7**

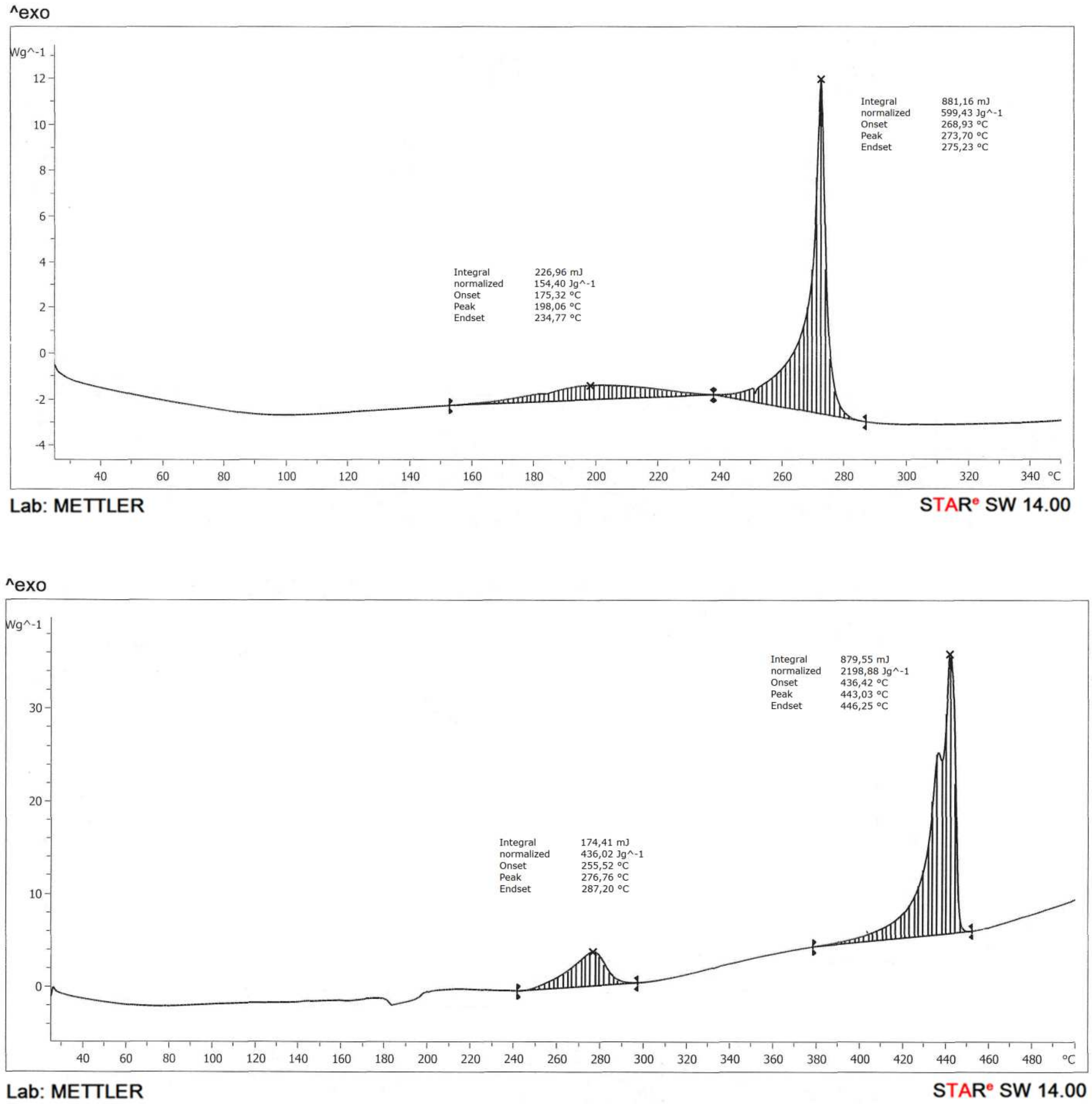


**Figure S5.** DSC of **8**

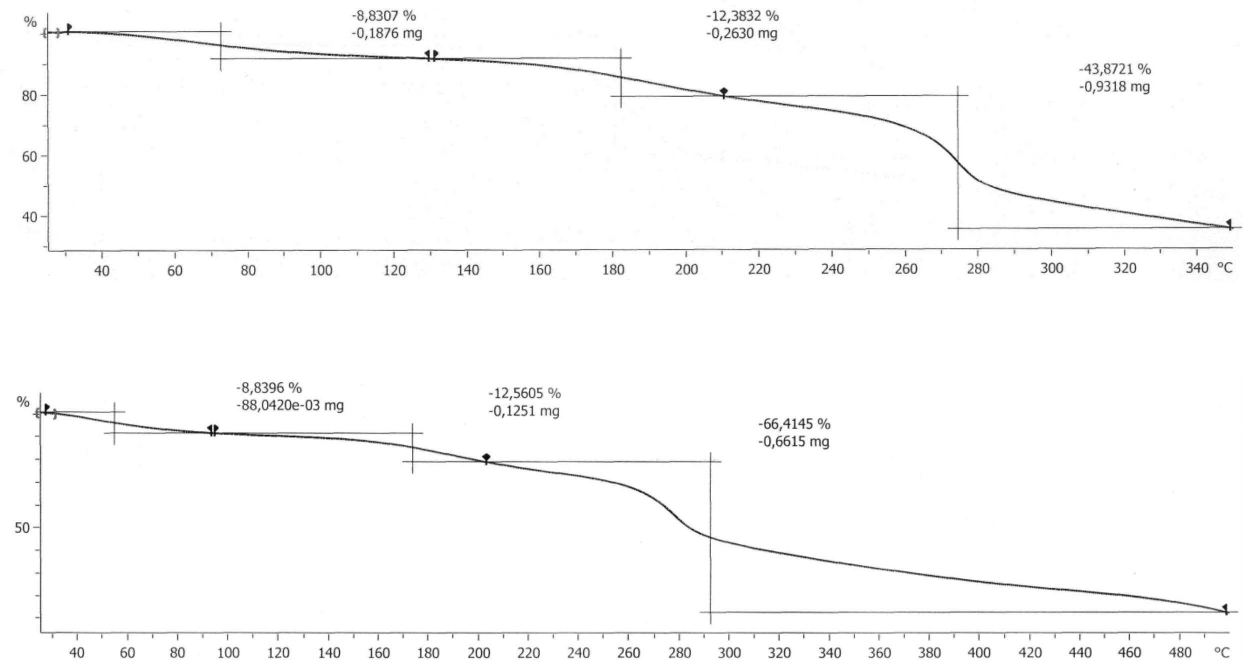


**Figure S6.** TGA of **8**

## Testing compound 8 for burning rate of $KNO_3$/Zr pyrotechnic composition

The following formulation was employed as the model composition: 52% zirconium (PCZr-1 grade (powdered calciothermic Zr), technical specifications No. 48-4-234-84, particle size 1–15 µm) and 48% potassium nitrate (chemically pure grade, GOST R 4217-77, particle size 63–160 µm).

Compound **8** was used as the burning-rate modifier composed of $(MZ)_5$ $Ni[N(NO_2)_2]_2$ $10H_2O$ with added 3.71% Ni.

**Table S1.** Burning rate of model composition (no additives)

| Sample #, sensor # | *l*, mm | time, s | *u*,mm/s | mean *u*, mm/s |
|---|---|---|---|---|
| 1.1-2 | 4.28 | 0.11864 | 36,07552 | 33.9783 |
| 1.2-3 | 4.40 | 0.11967 | 36,76778 | |
| 1.3-4 | 4.34 | 0.12108 | 35,84407 | |
| 2.1-2 | 4.40 | 0.12851 | 34,23858 | |
| 2.2-3 | 4.22 | 0.12901 | 32,71140 | |
| 2.3-4 | 4.18 | 0.13071 | 31,97944 | |
| 3.1-2 | 4.40 | 0.12910 | 34,08211 | |
| 3.2-3 | 4.22 | 0.14007 | 30,12779 | |
| 3.3-4 | 4.24 | * failure | - | |

Charge density: 2.82–2.95 g/cm$^3$.

* failure – simultaneous short circuit of sensors no.3 and no.4.

**Table S2.** Burning rate with 0.5% **8**

| Sample #, sensor # | *l*, mm | time, s | *u*, mm/s | mean *u*, mm/s |
|---|---|---|---|---|
| 1.1-2 | 4.3 | 0.18004 | 23.88358 | 22.6255 |
| 1.2-3 | 4.2 | 0.18543 | 22.65006 | |
| 1.3-4 | 4.38 | 0.22208 | 19.72262 | |
| 2.1-2 | 4.32 | 0.17777 | 24.30065 | |
| 2.2-3 | 4.36 | 0.19379 | 22.49858 | |
| 2.3-4 | 4.22 | 0.20021 | 21.07787 | |
| 3.1-2 | 4.36 | 0.19331 | 22.55445 | |
| 3.2-3 | 4.32 | 0.17367 | 24.87476 | |
| 3.3-4 | 4.22 | 0.19124 | 22.06651 | |

Charge density: 2.76–2.84 g/cm$^3$.

**Table S3.** Burning rate with 1.0% **8**

| Sample #, sensor # | *l*, mm | time, s | *u*, mm/s | mean *u*, mm/s |
|---|---|---|---|---|
| 1.1-2 | 4.3 | 0.19871 | 21.63958 | 21.4868 |
| 1.2-3 | 4.3 | 0.20220 | 21.26607 | |
| 1.3-4 | 4.3 | 0.19406 | 22.15810 | |
| 2.1-2 | 4.32 | 0.21564 | 20.03339 | |
| 2.2-3 | 4.24 | 0.17506 | 24.22027 | |
| 2.3-4 | 4.28 | 0.22028 | 19.42982 | |

| | | | | |
|---|---|---|---|---|
| 3.1-2 | 4,22 | 0.19612 | 21.51744 | |
| 3.2-3 | 4,22 | 0.20582 | 20.50335 | |
| 3.3-4 | 4,24 | 0,18750 | 22,61333 | |

Charge density: 2.81–2.91 g/cm$^3$.

**Table S4.** Burning rate with 1.5% **8**

| Sample #, sensor # | *l*, mm | time, s | *u*, mm/s | mean *u*, mm/s |
|---|---|---|---|---|
| 1.1-2 | 4.22 | 0.20375 | 20.71166 | 20.0348 |
| 1.2-3 | 4.3 | 0.18301 | 23.49598 | |
| 1.3-4 | 4.34 | 0.18423 | 23.55751 | |
| 2.1-2 | 4.54 | 0.23788 | 19.08525 | |
| 2.2-3 | 4.26 | 0.25301 | 16.83728 | |
| 2.3-4 | 4.18 | 0.22551 | 16.52109 | |
| 3.1-2 | 4.34 | *failure | | |
| 3.2-3 | 4.64 | *failure | | |
| 3.3-4 | 4.4 | *failure | | |

Charge density: 2.81–2.87 g/cm$^3$.
* failure – simultaneous triggering of sensors nos.1, 2 and 4.

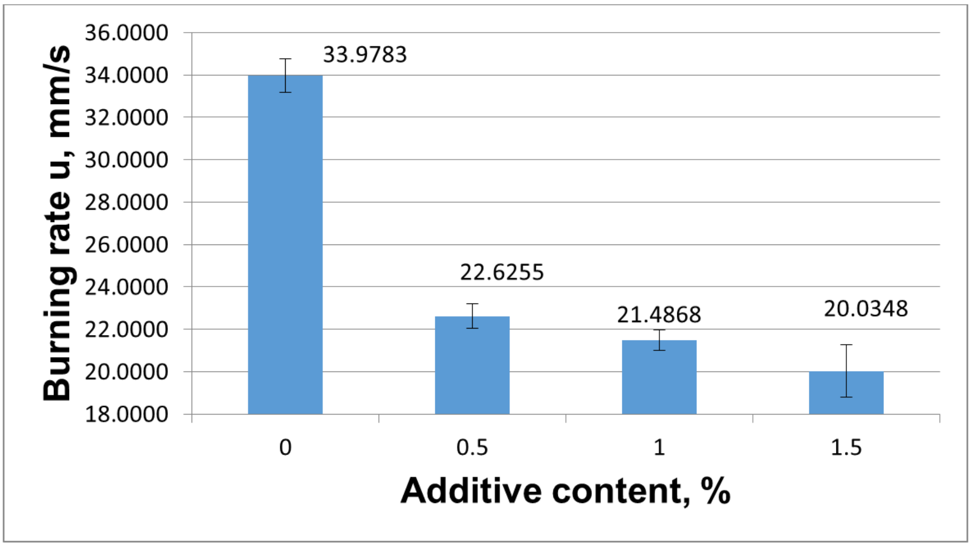


**Figure S7.** Effect of modifier **8** on the variation in the burning rate of $KNO_3$/Zr

## Testing compound 8 for burning rate of $KClO_4$/Al pyrotechnic composition

The following formulation was employed as the model composition: 70% potassium perchlorate (chemically pure grade, technical specifications No. 6-09-3801-76, particle size 63–160 µm) and 30% aluminum (ASD-4 grade, technical specifications No. 48-5-226-87, particle size 4–10 µm).

Compound **8** was used as the burning-rate modifier composed of $(MZ)_5$ $Ni[N(NO_2)_2]_2$ $10H_2O$ with added 3.71% Ni.

**Table S5.** Burning rate of model composition (no additives)

| Sample #, sensor # | *l*, mm | time, s | *u*, mm/s | mean *u*, mm/s |
|---|---|---|---|---|
| 1.1-2 | 5.22 | 1.31879 | 3.958174 | 3.857847 |
| 1.2-3 | 5.3 | 1.12713 | 4.702208 | |
| 1.3-4 | 5.32 | *failure | | |
| 2.1-2 | 5.36 | 1.42547 | 3.760163 | |
| 2.2-3 | 5.4 | 1.75731 | 3.072878 | |
| 2.3-4 | 5.34 | 1.45117 | 3.679789 | |
| 3.1-2 | 5.34 | 1.42106 | 3.757758 | |
| 3.2-3 | 5.28 | 1.49087 | 3.541556 | |
| 3.3-4 | 5.44 | 1.23911 | 4.390248 | |

Charge density: 2.27–2.35 g/cm$^3$.
* failure – no short circuit of sensor no.4.

**Table S6.** Burning rate with 0.5% **8**

| Sample #, sensor # | *l*, mm | time, s | *u*, mm/s | mean *u*, mm/s |
|---|---|---|---|---|
| 1.1-2 | 5.34 | 1.5716 | 3.3978 | 2.9385 |
| 1.2-3 | 5.4 | 1.6919 | 3.1917 | |
| 1.3-4 | 5.52 | 0.7069 | 7.8093 | |
| 2.1-2 | 10.88 | 4.2119 | | |
| 2.2-3 | 5.36 | *failure | | |
| 2.3-4 | 5.3 | 0.5734 | 9.2439 | |
| 3.1-2 | 5.42 | 1.8986 | 2.8547 | |
| 3.2-3 | 5.38 | 1.8833 | 2.8567 | |
| 3.3-4 | 5.3 | 1.9293 | 2.7471 | |

Charge density: 2.28–2.33 g/cm$^3$.
* * failure – simultaneous short circuit of sensors nos. 2 and 3

**Table S7.** Burning rate with 1.0% **8**

| Sample #, sensor # | *l*, mm | time, s | *u*, mm/s | mean *u*, mm/s |
|---|---|---|---|---|
| 1.1-2 | 5.38 | 4.1184 | 1.3063 | 2.3835 |
| 1.2-3 | 5.42 | 2.4752 | 2.1897 | |
| 1.3-4 | 5.40 | 1.7472 | 3.0907 | |

| | | | | |
|---|---|---|---|---|
| 2.1-2 | 5.36 | 5.3675 | 0.9986 | |
| 2.2-3 | 5.38 | 1.9157 | 2.8084 | |
| 2.3-4 | 5.42 | 1.3289 | 4.0786 | |
| 3.1-2 | 5.40 | 3.6334 | 1.4862 | |
| 3.2-3 | 5.50 | 2.3696 | 2.3211 | |
| 3.3-4 | 5.40 | 1.7026 | 3.1716 | |

Charge density: 2.25–2.3 g/cm$^3$.

**Table S8.** Burning rate with 1.5% **8**

| Sample #, sensor # | *l*, mm | time, s | *u*, mm/s | mean *u*, mm/s |
|---|---|---|---|---|
| 1.1-2 | 5.26 | 1.6196 | 3.2477 | 2.6643 |
| 1.2-3 | 5.42 | 2.2380 | 2.4218 | |
| 1.3-4 | 5.22 | 0.6380 | 8.1814 | |
| 2.1-2 | 5.5 | 1.8686 | 2.9434 | |
| 2.2-3 | 5.28 | 2.8244 | 1.8694 | |
| 2.3-4 | 5.34 | 1.4407 | 3.7065 | |
| 3.1-2 | 5.34 | 4.1069 | 1.3003 | |
| 3.2-3 | 5.28 | 2.8503 | 1.8524 | |
| 3.3-4 | 5.54 | 1.3945 | 3.9728 | |

Charge density:2.28–2.32 g/cm$^3$.

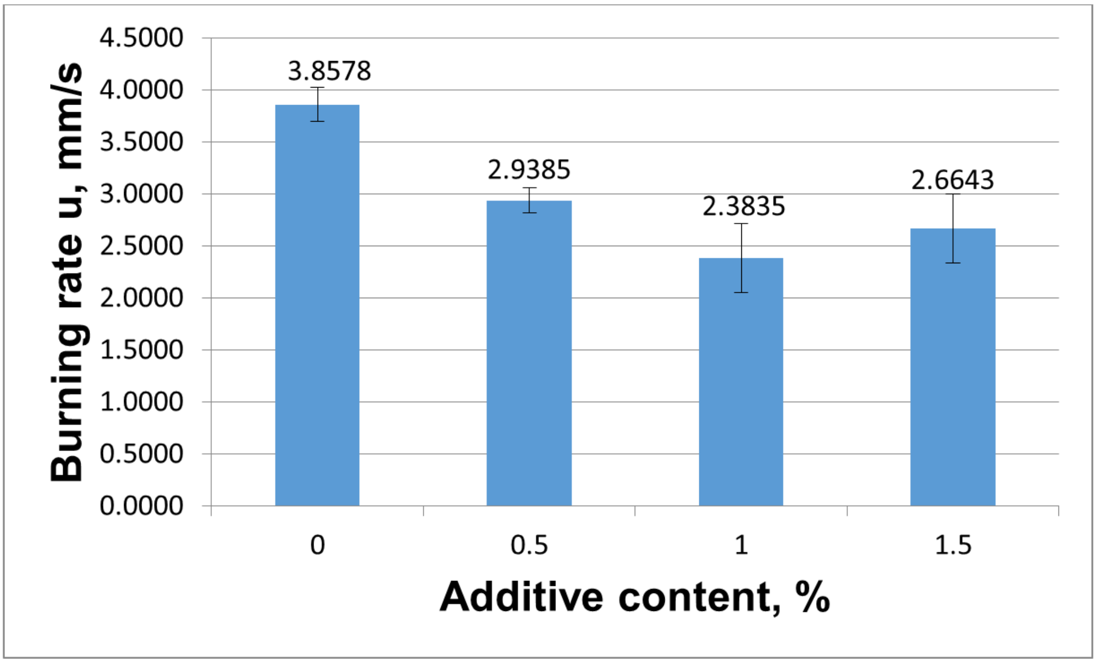


**Figure S8.** Effect of **8** on the burning rate of the $KClO_4$/Al pyrotechnic composition
